# Are Disability Rates among People with Diabetes Increasing in Germany? A Decomposition Analysis of Temporal Change between 2004 and 2015

**DOI:** 10.1177/0898264320970324

**Published:** 2020-10-31

**Authors:** Stefanie Sperlich, Johannes Beller, Jelena Epping, Batoul Safieddine, Juliane Tetzlaff, Siegfried Geyer

**Affiliations:** 1 Medical Sociology, 9177 Hannover Medical School, Hannover, Germany

**Keywords:** diabetes, functioning, disability, elderly, health status, temporal change

## Abstract

**Objectives:** We investigated changes in the prevalence of disabilities among individuals with type 2 diabetes and analyzed the contribution of comorbidities on this change. **Methods:** Data were drawn from the Survey of Health, Ageing, and Retirement in Europe (SHARE). We estimated predicted probabilities of impaired (instrumental) activities of daily living (IADL and ADL) by means of logistic regression. Multivariate decomposition was employed for analyzing the impact of comorbidities on changes in disability rates. **Results:** Among people with diabetes, ADL difficulties rose significantly from 11.3% (2004) to 19.1% (2015), while IADL difficulties increased among younger diabetics from 11.5% to 18.3%. Decomposition analysis revealed that the parallel increase in comorbidities contributed to the rise in disabilities. **Discussion:** We found disability rates among people with diabetes in Germany to be increasing over time, pointing toward a growing demand of tertiary prevention for these individuals to maintain functional health and quality of life.

## Background

Different hypotheses have been proposed to describe the dynamics of health changes in the population within the context of increasing life expectancy. While Fries’ hypothesis of “morbidity compression” assumes that life years spent in states of morbidity will decrease (Fries, 1980), the “morbidity expansion” hypothesis posits that increased lifetime will entail an increase in the number of years spent in states of disease and disability (Gruenberg, 2005). A third hypothesis, the “dynamic equilibrium” postulates that longer survival is associated with an increase of life years in morbidity, but due to medical advances and healthier lifestyles, time spent in severe disability will decline (Manton, 1982). This assumption implies that individuals are able to master everyday life increasingly well in spite of chronic conditions.

For the case of type 2 diabetes, evidence suggests a marked increase of prevalence rates over the past decades. It is globally estimated that between 1980 and 2014, age-standardized prevalence among adult men doubled and increased by 60% in women (Krug, 2016). These trends were accompanied by large reductions of mortality rates leading to an increasing number of years spent with diabetes (Rowley et al., 2017). In Germany, the current diabetes prevalence is estimated to range between 7.2% and 9.9% (Heidemann, 2017). The projection of number of future type 2 diabetes cases indicates a relative increase in the number of diabetes cases of between 54% and 77% from 2015 to 2040 (Tönnies et al., 2019).

This development of rising prevalence rates of type 2 diabetes and the simultaneous increase in life expectancy that was also observed among people with diabetes (Muschik et al., 2017) clearly contradicts the assumption of morbidity compression. However, in order to decide whether “morbidity expansion” or “dynamic equilibrium” applies, additional information on trends of diabetes-related disabilities is required. A dynamic equilibrium would be the case if the diabetes increase may be partially offset by a shift from major to moderate disabilities. Monitoring such information on diabetes-related disability in addition to the incidence and prevalence rates of diabetes seems to be critical for healthcare policy and planning related to diabetes management.

Previous studies have shown a greater risk of disability among people with diabetes than individuals without this condition (Wong et al., 2013). Multimorbidity, defined as the occurrence of two or more diseases within a person, is associated with a greater likelihood of disability and reduced health-related quality of life than single disease (Sheridan et al., 2019). For older adults with diabetes, having at least one additional concurrent chronic condition is common (Magnan et al., 2018). The most frequent comorbidities of diabetes are hypertension, overweight or obesity, hyperlipidemia, chronic kidney disease, and cardiovascular disease (Iglay et al., 2016). Comorbidities together with poor glycemic control turned out to explain a substantial part of the elevated disability rates associated with diabetes (Kalyani et al., 2010). In addition, depression is a frequently occurring comorbidity that leads to impaired ability for the self-management of the disease (Bo et al., 2019).

The growing relevance of diabetes worldwide has motivated further research to improve the management of patients with diabetes. Like in other countries, disease management programs (DMPs) were introduced in Germany in order to improve the quality of health care and the treatment process. Recent studies analyzing disability trends in the diabetic population point to decreasing rates of complications and functional limitations; however, respective studies are rather scarce (Bardenheier et al., 2016; Gregg et al., 2014; Nowakowska et al., 2019; Rawshani et al., 2017). Against this backdrop, we aimed to investigate the temporal change of disability prevalence among people with diabetes in Germany by taking changes in comorbidity into account. More specifically, the study is guided by the following research questions:

1. Has the prevalence of type 2 diabetes increased between the years 2004 and 2015?
2. How did the disability prevalence in terms of activities of daily living (ADL) and instrumental activities of daily living (IADL) change in people with type 2 diabetes as compared with people without this chronic condition?
3. Does increasing comorbidity prevalence (somatic comorbidities, obesity, and depression) account for changes in disability prevalence among people with diabetes?

## Methods

### Sample

Data were derived from the Survey of Health, Ageing and Retirement in Europe (SHARE). SHARE is a cross-national, longitudinal, and population-based survey of noninstitutionalized Europeans conducted in 28 countries. The target population consists of all persons aged 50 years and above at the time of sampling. Persons who are hospitalized or unable to speak the country’s language were excluded. Computer-assisted personal interviewing was used during face-to-face interviews to collect information on health, socioeconomic conditions, psychosocial factors, and social networks. Respondents with physical or cognitive limitations could be assisted by a proxy respondent. Further details on study design and sampling methods have been published by the study’s authors (Börsch-Supan et al., 2013). Our analyses referred to Germany and included individuals over 49 years of age. We used waves 1 and 6 from the panel survey. The intermediate waves were not analyzed as we aimed to compare distinct, nonoverlapping samples avoiding counting diabetes in the same subject several times. For this purpose, we furthermore dropped all subjects in wave 6 that have already participated in wave 1. With this approach, we also minimized sample bias that might be caused by selective survey participation. Data collection in wave 1 partly extended to 2005. We used calibrated cross-sectional individual weights for ensuring a high degree of representativeness for the German population. The weights are based on calibration margins for the size of the target population across eight gender–age-groups and major socio-economic regions according to level 1 of NUTS (Nomenclature des unités territoriales statistiques). Overall, 2918 respondents in wave 1 (1363 men/1555 women) and 3637 in wave 6 (1738 men/1899 women) were included. Respondents with missing information on the variables used for analysis were excluded.

### Measures

#### Identification of type 2 diabetes cases

Respondents were asked about chronic conditions via face-to-face interviews by asking the following question: “Has a doctor ever told you that you have any of the conditions on this card?” Persons who indicated “yes” to the condition “diabetes or high blood sugar level” were classified as having diabetes. After confirming a chronic condition, participants were further asked about the disease’ age of onset by asking: “About how old were you when the doctor said you had this condition?” This information was used to identify possible cases of type 1 diabetes, as our aim was to analyze temporal changes in individuals with type 2 diabetes. In case of type 2 diabetes, the pancreas produces insulin, but the body cannot use it effectively. By contrast, in case of type 1 diabetes, the pancreatic beta cells no longer produce insulin at all. While type 1 diabetes usually appears first in children and adolescents, type 2 diabetes is more prevalent in older people. Correct diagnosis of type 1 diabetes in young people is usually not difficult because it accounts for most cases of diabetes in that population. By contrast, in older adults above 30 years of age, newly diagnosed type 1 diabetes cases are rare, accounting for less than 5% of all diabetes cases (Thomas et al., 2018). Based on these distributions, we classified those with age at onset below 31 years as type 1 diabetes and excluded these cases from our analysis (n=26 out of 837 diabetic cases in total).

#### Disability assessment: ADL and IADL

Disability is measured by assessing difficulties of ADL (Katz, 1983) as well as IADL (Lawton & Brody, 1969). The ADL index refers to very basic everyday self-care activities such as dressing, walking, bathing, eating, and toileting, which are fundamental for maintaining independence. The IADL index (Lawton & Brody, 1969) describes the number of difficulties with more complex activities, such as managing money, going shopping, using a telephone, taking medication, or doing house chores. The modified version used in SHARE includes six activities of ADL and seven activities of IADL. In wave 6, the measure of IADL includes two additional activities (leaving the house independently and accessing transportation services/doing personal laundry). Thus, the resulting score ranges from 0 to 6 (ADL) and from 0 to 7/9 (IADL), respectively. SHARE provides also dichotomous categorical variables reclassifying ADL as well as IADL in two categories: 0 “no difficulty” and 1 “1+ difficulties.” We used the dichotomous variables since the frequency of more than one difficulty among persons with diabetes was only 10.5% for ADL and 15.9% for IADL. In addition, we assumed the dichotomous variable to be less sensitive to the change in IADL scoring that has taken place between waves.

#### Comorbidity assessment: somatic comorbidity, depression, and obesity

The presence of at least two additional chronic somatic conditions among individuals with diabetes was defined out of the following list of self-reports of diagnoses communicated by a doctor: myocardial infarction, high blood pressure, high cholesterol levels, stroke, cancer, stomach ulcers, lung disease (excluding asthma), Parkinson’s diagnoses, cataract, femoral neck, or hip fracture.

The EURO-D scale (Prince et al.,1999) was used for measuring depression. The scale consists of the following 12 items: depressed mood, pessimism, suicidality, guilt, sleep, interest, irritability, appetite, fatigue, concentration (on reading or entertainment), enjoyment, and tearfulness. The maximum score a respondent can get is 12, meaning “very depressed” and the minimum score is 0 “not depressed.” The attainment of a scale score of 4 or higher is categorized as “case of depression” and a scale score below 4 as “not depressed.” The generated dichotomous variable “eurodcat” has the value of 1 if the scale score is 4 or higher.

Body weight and height were orally assessed during the interview and the body mass index (BMI) was calculated from these two values using the following formula: weight in kg/height in m^2^. According to the standard categories determined by the World Health Organization (WHO, 1995), those with a BMI ≥ 30 were classified as having obesity.

#### Temporal Change

Change between time points was measured with a dichotomous variable with the categories 0 for wave 1 (2004) and 1 for wave 6 (2015). This variable was used as an independent variable for analyzing temporal changes in the prevalence of type 2 diabetes as well as disability.

### Statistical Analysis

We estimated predicted probabilities of self-reported diabetes in men and women for each wave by means of logistic regression analyses adjusted for age and education. Based on the same regression model, we estimated the odds of having at least one difficulty in terms of ADL and IADL in people with diabetes as compared with the nondiabetic population, stratified by gender and wave and adjusted for age and education. In addition to odds ratio, we reported predicted probabilities of disabilities adjusted for age and education, calculated by setting each confounder to its mean value. With this approach, the mean age of both genders was held constant for both waves. Change in ADL and IADL proportions among persons with diabetes were reported separately for “younger olds” (50–69 years) and older ages (70 years and above).

Multivariate decomposition for nonlinear response models (Yun et al., 2011) was employed for the third research question of whether rise in somatic comorbidities as well as in obesity and depression rates between waves 1 and 6 may account for changes in ADL and IADL proportions among individuals with diabetes. The technique uses the output from logit regression models for partitioning change over time into components attributable to changing characteristics (*E*) and to changing effects (*C*). By applying this procedure, the observed difference in proportions of ADL and IADL difficulties between waves 1 and 6 will be additively decomposed into these two components. The component labeled ‘E’ refers to the part of the change attributable to differences in endowments or characteristics between waves 1 and 6, usually called the explained component or characteristics. In our study, that would be the temporal change in the number of persons with diabetes affected by somatic comorbidities, obesity, and depression. By contrast, the component ‘C’ refers to the part of the differential attributable to differences in coefficients or effects, usually called the unexplained component or coefficient effects. In our case, this would be a shift in the effect size of somatic comorbidities, obesity, and depression on ADL and IADL difficulties.

Wave 6 was chosen as the reference group, thus *E* reflects a counterfactual comparison of the differences in outcomes from wave 6 perspective (that is, the expected difference in ADL and IADL difficulties between waves 1 and 6 among individuals with diabetes if wave 6 were given wave 1 distribution of covariates). By contrast, the *C* reflects a counterfactual comparison of outcomes from wave 1 perspective (that is, the expected difference if wave 1 would have the coefficients of wave 6). We decomposed the observed change in ADL and IADL difficulties among individuals with diabetes using a logit model with a set of predictors including age, somatic comorbidities, obesity, and depression. We applied the Stata command “mvdcmp” (Yun et al., 2011) for carrying out the multivariate decomposition which provides the detailed composition and standard errors for the characteristics component (change in the characteristics or endowments over time) and the coefficient component (change in the effect of predictors).

## Results

### Changes in Predicted Probabilities of Diabetes between Waves 1 and 6

Individuals with diabetes were older and had lower levels of education than those without diabetes (Table 1). Predicted probabilities of diabetes increased slightly from 10.9% in 2004 to 12.4% in 2015 (Table 2). Stratified by gender, it turned out that in men, predicted probability of diabetes rose significantly from 10.9% to 14.8%, while a slight decrease from 10.7% to 10.1% was observed in women. The rise in diabetes prevalence was most pronounced among men aging 70 years and above where predicted probabilities rose from 11.6% to 19.9%.

**Table 1.** Weighted Sample Characteristics in %.

	People with diabetes		People without diabetes	
	Wave 1 (*n* = 320)	Wave 6 (*n* = 485)	Wave 1 (*n* = 2598)	Wave 6 (*n* = 3152)
Gender				
Male	42.3	54.5	45.6	46.4
Female	57.7	45.5	54.4	53.6
Missing (*n*)	0	0	0	0
Mean age				
Male	65.5	68.5	63.8	64.5
Female	70.1	69.5	65.8	65.7
Missing (*n*)	0	0	0	0
Age-group (years)				
50–69	55.5	50.8	68.9	67.7
70 +	44.5	49.2	31.1	32.3
Missing (*n*)	0	0	0	0
Education				
Primary	28.2	19.5	17.1	11.8
Secondary	54.3	55.5	56.2	57.8
Tertiary	17.6	25.5	26.8	30.5
Missing (*n*)	4	5	24	25

*Note.* Wave 1 = 2004; wave 6 = 2015; *n* = number of cases; standard deviation of mean age: men with diabetes wave 1/wave 6: 8.3/9.1, women with diabetes 9.6/10.0, men without diabetes: 9.5/9.7, women without diabetes 9.7/10.9, and SHARE-study Germany, 2004 and 2015.

**Table 2.** Change in Diabetes Prevalence between Wave 1 and Wave 6 Stratified by Gender.

	Overall			Men			Women		
	OR	95% CI	%	OR	95% CI	%	OR	95% CI	%
All ages									
Wave 6	1.15	.97–1.37	12.4	1.42**	1.12–1.80	14.8	.94	.73–1.21	10.1
Wave 1 (reference)	1		10.9	1		10.9	1		10.7
50–69 years									
Wave 6	1.11	.89–1.39	9.9	1.25	.93–1.68	12.7	.94	.68–1.31	6.8
Wave 1 (reference)	1		8.9	1		10.5	1		7.1
70+ years									
Wave 6	**1.28†**	.97–1.67	18.9	1.89**	1.26–2.85	19.9	1.01	.70–1.46	17.9
Wave 1 (reference)	1		15.4	1		11.6	1		17.7

*Note.* Adjusted for age and education, wave 1 = 2004; wave 6 = 2015; OR = odds ratio; % = predicted probabilities; *p < .05; **p < .01; ***p < .001; †*p* < .10; SHARE-study Germany, 2004 and 2015.

### Changes in ADL and IADL among Individuals with and Without Diabetes

**Figure 1.**
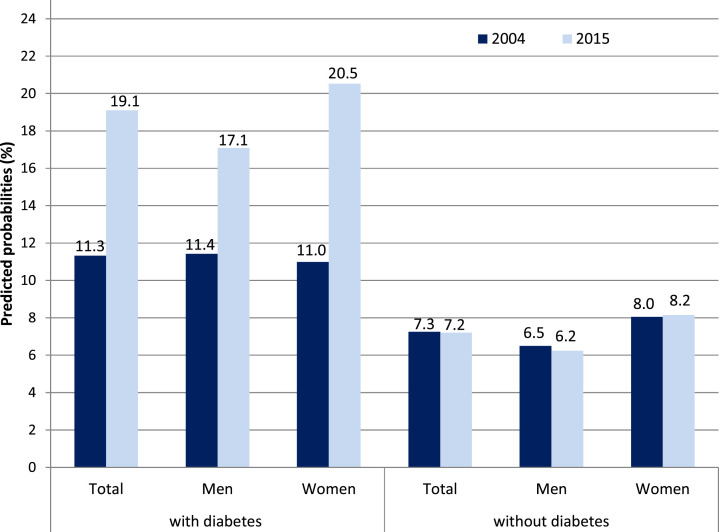
Change in ADL between 2004 and 2015 among people with and without diabetes, predicted probabilities in %. Adjusted for age and education, for the bars “total” also for gender, SHARE-study Germany, 2004 and 2015. *Note.* ADL = activities of daily living; SHARE = Survey of Health, Ageing, and Retirement in Europe.

**Figure 2.**
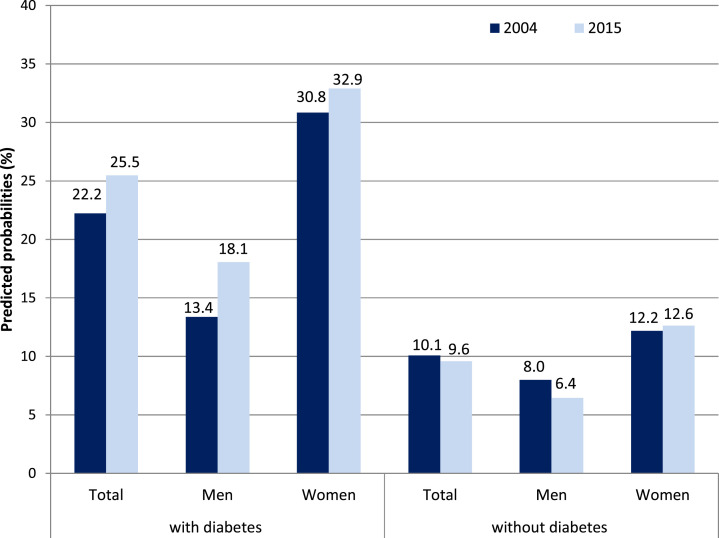
Change in IADL between 2004 and 2015 among people with and without diabetes, predicted probabilities in %. Adjusted for age and education, for the bars “total” also for gender, SHARE-study Germany, 2004 and 2015. *Note.* IADL = instrumental activities of daily living; SHARE = Survey of Health, Ageing, and Retirement in Europe.

Among individuals with type 2 diabetes, predicted probabilities of at least one difficulty in ADL significantly rose from 11.3% in 2004 to 19.1% in 2015 (Figure 1). The gender-stratified analyses showed that this holds for women and for men, while the effects were statistically significant in women only (Table 3). IADL difficulties increased only moderately among individuals with diabetes with a higher rise observed in men with diabetes. By comparing “younger olds” and “older ages,” it turned out that ADL difficulties rose more strongly for the latter. By contrast, IADL difficulties significantly increased for the “younger olds,” while they tended to decrease for those aged 70 years and above. Among individuals without diabetes, predicted probabilities of ADL and IADL difficulties remained almost unchanged over time for both genders (Figures 1 and 2).

**Table 3.** Change in ADL and IADL Prevalence between Wave 1 and Wave 6 among Men and Women with Diabetes.

	ADL								
	Overall			Men			Women		
	OR	95% CI	%	OR	95% CI	%	OR	95% CI	%
All ages									
Wave 6	1.85**	1.15–2.97	19.1	1.60	.82–3.12	17.1	2.09*	1.08–4.06	20.5
Wave 1 (reference)	1		11.3			11.4	1		11.0
50–69 years									
Wave 6	1.67	.79–3.55	12.6	1.45	.56–3.77	12.4	2.13	.59–7.72	12.8
Wave 1 (reference)	1		7.9	1		8.9	1		6.4
70+ years									
Wave 6	1.93*	1.02–3.65	28.5	1.63	.61–4.31	27.8	**1.97†**	.88–4.41	27.2
Wave 1 (reference)	1		17.1	1		19.2	1		15.9

	IADL								
	Overall			Men			Women		
	OR	95% CI	%	OR	95% CI	%	OR	95% CI	%
All ages									
Wave 6	1.20	.81–1.76	25.5	1.43	.72–2.64	18.1	1.10	.66–1.84	32.9
Wave 1 (reference)	1		22.2	1		13.4	1		30.8
50–69 years									
Wave 6	2.37**	1.32–4.28	18.3	1.84	.77–4.55	13.9	1.55	.73–3.28	25.6
Wave 1 (reference)	1		11.5	1		8.1	1		18.1
70+ years									
Wave 6	0.89	.50–1.58	36.6	0.99	.40–2.43	32.4	0.79	.38–1.63	37.1
Wave 1 (reference)	1		39.3	1		32.6	1		42.6

*Note.* Overall change adjusted for age, gender and education, change in men and women adjusted for age and education, wave 1 = 2004; wave 6 = 2015; OR = odds ratio; % = predicted probabilities; ADL = activities of daily living; IADL = instrumental activities of daily living; *p < .05; **p < .01; ***p < .001, †*p* < .10, SHARE-study Germany, 2004 and 2015.

### Changes in Characteristics and Coefficients of Comorbidity on ADL and IADL

Evaluating the differences in characteristics between waves 1 and 6 among men with diabetes revealed substantially higher levels of somatic comorbidities and a higher average age in wave 6 than wave 1 (Table 4). Also, obesity and depression prevalence increased, however, failing to reach statistical significance. Among women with diabetes, a clear rise in somatic comorbidities and obesity was found, while the change in depression prevalence was less pronounced.

**Table 4.** Change in Characteristics of Somatic Comorbidities, Obesity, Depression and Age between Wave 1 and Wave 6 among Men and Women with Diabetes.

	Men with diabetes											
	Somatic comorbidities			Obesity			Depression			Age		
	OR	95% CI	%	OR	95% CI	%	OR	95% CI	%	Coef	95% CI	Mean
Wave 6	**1.54†**	.97–2.44	46.8	1.11	.70–1.76	41.4	1.19	.67–2.13	23.5	3.08**	1.32–4.83	68.5
Wave 1 (reference)	1		36.4	1		38.8	1		20.5	1		65.5

	Women with diabetes											
	Somatic comorbidities			Obesity			Depression			Age		
	OR	95% CI	%	OR	95% CI	%	OR	95% CI	%	Coef.	95% CI	Mean
Wave 6	2.32***	1.45–3.70	58.6	2.26**	1.39–3.63	50.9	1.39	0.86–2.26	42.0	−.67	−2.67–1.33	69.5
Wave 1 (reference)	1		37.9	1		31.5	1		34.2	1		70.1

*Note.* Adjusted for age and education, wave 1 = 2004; wave 6 = 2015; somatic comorbidities = at least two additional somatic comorbidities from a list of 10 chronic somatic diseases; % = predicted probabilities; mean = predicted mean age; OR = odds ratio; Coef. = coefficient; 95% CI = 95% confidence interval; *p < .05; **p < .01; ***p < .001; †*p* < .10.

Analyzing changes in the effect size of these comorbidities revealed that the impact of obesity and depression on ADL and IADL in men with diabetes rose significantly from wave 1 to wave 6 (Table 5). Among women with diabetes, no changes in coefficients were observed for depression where effects on ADL and IADL revealed to be significant in both waves. Similarly, no change in coefficients occurred for obesity, while the effect of somatic comorbidities on ADL and IADL tended to increase.

**Table 5.** Effects of Somatic Comorbidities, Obesity, Depression and Age on ADL and IADL in Wave 1 and Wave 6 among Men and Women with Diabetes.

	ADL				IADL			
	Men		Women		Men		Women	
	OR	95% CI	OR	95% CI	OR	95% CI	OR	95% CI
Somatic comorbidities								
Wave 1	1.98	.61–6.42	1.17	.37–3.68	1.12	.34–3.64	.61	.26–1.46
Wave 6	1.84	.86–3.92	1.74	.70–4.34	1.29	.61–2.73	1.73	.80–3.71
Obesity								
Wave 1	2.28	.68–7.66	1.41	.43–4.65	1.67	.62–4.52	1.30	.54–3.15
Wave 6	2.80**	1.31–5.97	1.29	.54–3.09	2.96**	1.32–6.64	1.20	.55–2.60
Depression								
Wave 1	**3.60†**	.91–14.2	3.62*	1.23–10.65	2.74	.76–9.92	3.51**	1.51–8.14
Wave 6	3.17**	1.37–7.33	2.70*	1.07–6.81	5.24***	2.25–12.34	2.38*	1.11–5.12

Age	Coef.	95% CI	Coef.	95% CI	Coef.	95% CI	Coef.	95% CI
Wave 1	1.05	.97–1.14	1.00	.93–1.08	1.10*	1.01–1.20	1.06*	1.01–1.13
Wave 6	1.07**	1.02–1.13	1.08**	1.02–1.13	1.11***	1.06–1.17	1.03	0.99–1.08

*Note.* Multivariate model adjusted for age and education, wave 1 = 2004; wave 6 = 2015; somatic comorbidities = at least two additional somatic comorbidities from a list of 10 chronic somatic diseases; OR = odds ratio; 95% CI = 95% confidence interval; Coef. = coefficient; ADL = activities of daily living; IADL = instrumental activities of daily living; *p < .05; **p < .01; ***p < .001; †*p* < .10.

### Decomposition Analyses

Carrying out the decomposition analyses, we found the proportion of ADL difficulties increased in total by 5.9% points from wave 1 to 6 among men with diabetes (Table 6). *Changing characteristics* accounted for 4.5 % points, while *changing effects* accounted for 1.4% points of this increase. Changes in the age composition (Table 4) contributed most to the increase in ADL difficulties due to changing characteristics (2.7% points). In addition, the increase in obesity and depression prevalence (Table 4) significantly accounted for rising ADL difficulties in wave 6 (both with 0.3% points). With respect to *changing coefficients*, no significant shift in the effect of any of the comorbidities was observed. For IADL decomposition, the picture among men was similar, showing that the total difference between waves 1 and 6 (5.4% points) was mainly due to changing characteristics accounting for 5.3% points of the increase in IADL difficulties. Again, this change was mainly due to a shift in age composition (3.9% points). In addition, increases in obesity and depression levels also significantly contributed to the rise in IADL difficulties.

**Table 6.** Decomposition of the Difference in ADL/IADL between Wave 1 and Wave 6 into Components Attributable to Changing Characteristics and Changing Coefficients.

	Men					
	ADL			IADL		
	Coef. (%)	95% CI	Pct.	Coef. (%)	95% CI	Pct.
Difference in total	**5.9†**	−.5–12.3	100	5.4	−.1–12.2	100
Due to difference in E	4.5***	2.6–6.4	76.1	5.3***	3.3–7.2	97.3
Due to difference in C	1.4	−4.7–7.5	23.9	.1	−6.4–6.7	2.7
	Due to difference in characteristics (E)					
Somatic comorbidities	1.1	−.2–2.5	19.0	.5	−.8–1.8	9.1
Obesity	.3*	.0–.6	5.7	.4**	.1–7.1	7.8
Depression	.3*	.0–.6	5.5	.5***	.2–7.4	9.0
Age	2.7**	1.2–4.3	45.9	3.9***	2.4–5.4	71.4
	Due to difference in coefficients (C)					
Somatic comorbidities	.8	−3.0–4.5	12.8	−.7	−37.3–35.9	−12.5
Obesity	−.2	−2.4–2.8	−4.1	−.8	−43.3–41.8	−14.5
Depression	−.8	−3.0–.9	−12.8	−.2	−10.3–10.0	−3.5
Age	−.4	−8.8–7.9	−7.2	.1	−8.3–8.5	2.4
Intercept	2.1	−10.6–14.7	35.1	1.7	−8.6–9.0	30.7

	Women					
	ADL			IADL		
	Coef. (%)	95% CI	Pct.	Coef. (%)	95% CI	Pct.
Difference in total	**5.8†**	−.9–12.5	100	−4.1	−.4–12.2	100
Due to difference in E	1.0	−1.3–3.4	17.5	+2.8*	.4–5.2	−68.6
Due to difference in C	4.8	−2.1–11.7	82.5	−6.9	−15.6–1.8	168.6
	Due to difference in characteristics (E)					
Somatic comorbidities	.6	−0.9–2.0	10.0	1.9*	0.2–3.6	−47.3
Obesity	.7	−1.0–2.4	11.8	.7	−1.0–2.5	−18.2
Depression	.6***	.3–0.9	10.4	.8***	.4–1.2	−18.9
Age	−0.8*	−15.6- −0.1	−14.6	−.6*	−1.3 - −0.1	15.8
	Due to difference in coefficients (C)					
Somatic comorbidities	2.2	−8.2–12.6	37.7	8.8*	.8–16.9	−216.1
Obesity	.5	−8.6–9.5	7.9	−1.5	−9.3–6.3	36.8
Depression	.9	−8.5–10.2	14.9	−1.3	−8.8–6.1	32.8
Age	15.9	−36.4–68.1	273.8	−21.8	−57.0–13.3	532.8
Intercept	−14.6	−82.5–53.3	−251.8	8.9	−30.8–48.7	−217.8

*Note.* wave 1 = 2004; wave 6 = 2015; somatic comorbidities = at least two additional somatic comorbidities from a list of 10 chronic somatic diseases; E = Characteristics; C = coefficients; Coef. (%) = coefficients multiplied with 100; CI = confidence interval; Pct. = expressed as percentage; ADL = activities of daily living; IADL = instrumental activities of daily living; ***p < .05; **p < .01; ***p < .001; †*p* < .10, SHARE-study Germany, 2004 and 2015.

Among women with diabetes, the prevalence of ADL difficulties increased by 5.8% points from wave 1 to wave 6. Unlike men with diabetes, the change in coefficients with 4.8% points accounted the most for this increase. However, each coefficient did not reach statistical significance. In contrast, the rise in ADL difficulties would be significantly reduced by 0.6% points when shifting the depression levels from wave 1 to wave 6. Shifting the age composition from wave 1 to wave 6 would lead to the opposite effect of rising ADL difficulties by 0.8% due to higher average age in wave 1 than wave 6 (Table 4). With respect to IADL, we found a total decrease in difficulties by 4.1% points, whereby this value is comprised of two opposing trends. Differences due to change in *characteristics* point to a significant increase by 2.8% points, while those due to change in *coefficients* indicate a decrease in IADL difficulties by 6.9% points. This decrease was mainly due to a weakening age effect from wave 1 to wave 6; however, this effect was not statistically significant. By contrast, the effect of somatic comorbidities on IADL difficulties tended to increase between waves 1 and 6 (Table 5), thus shifting this coefficient from wave 6 to wave 1 would provide a significant further decrease in IADL difficulties by 8.8% points. With regard to change in characteristics, IADL difficulties would increase by 0.6% points from 2.8% points to 3.4% points when shifting the age characteristics from wave 1 to wave 6. By contrast, shifting the characteristics of depression and somatic comorbidities from wave 1 to wave 6 would provide a significant decrease in IADL difficulties by 0.8% points and 1.9% points, respectively.

## Discussion

In light of the global trend of increasing prevalence of chronic conditions, the “dynamic equilibrium hypothesis” provides an optimistic scenario of the future development of population health. It states that the increase in diabetes prevalence may be partially offset by a shift from major to moderate disabilities. The accuracy of this assumption has far-reaching consequences for the future course of the global burden of diabetes to public health systems, as well as for the further development of health-related quality of life in people with this chronic condition.

We tested this thesis and found in line with the global trend a rise in diabetes prevalence between 2004 and 2015. However, the increase was restricted to men, while prevalence remained largely stable among women. The rise in type 2 diabetes can be principally explained by the secular trend of increasing overweight and obesity rates caused by the expansion of high-energy diet and sedentary lifestyle (Meldrum et al., 2017). In addition, also changes in diagnostic criteria and improvements in diabetes-related diagnostics were held responsible for this development (Heidemann, 2017). Supporting this assumption, the study by Heidemann and colleagues (Heidemann et al., 2016) revealed that the prevalence of diagnosed diabetes increased in Germany between 1997–1999 and 2008–2011, whereas the prevalence of undiagnosed diabetes decreased at the same time period. This finding suggests that at least a part of the rise in the prevalence of diagnosed type 2 diabetes may be due to improvements in the early detection of diabetes that had led to a shift in the proportion from undiagnosed to diagnosed diabetes.

### Change in ADL and IADL Difficulties According to Diabetes Status

Previous studies analyzing disability trends in the elderly point to decreasing rates of functional limitations (Aijänseppä et al., 2005; Feng et al., 2013; Schoeni et al., 2008; Trachte et al., 2015). Likewise, studies on disability trends among people with type 2 diabetes suggest a decrease of diabetes-related impairments and comorbidities. For example, Bardenheier et al. (2016) found that regardless of diabetes status, US adults experienced compression of disability and gains in disability-free life years. A British longitudinal study revealed decreasing rates of depression, hypertension, and asthma among people with diabetes between 2007 and 2017 (Nowakowska et al., 2019). In addition, Gregg et al. (2014) reported diabetes-related complications to be decreasing in the Unites States. Pointing toward the same direction, in a Swedish longitudinal study, Rawshani et al. (2017) found cardiovascular mortality among individuals with diabetes to decrease more substantially as compared with people without this condition. In contrast, Martinez-Huedo et al. (2011) reported an increasing prevalence of disability among people with diabetes in Spain between the years 2000 and 2007. In line with the latter finding, we found rising ADL disability among people with diabetes in Germany, while changes in IADL-related disability were less pronounced. These findings suggest that in particular rates of more severe disability among individuals with diabetes increased over time. However, we also found a significant increase in IADL difficulties in younger persons with diabetes, while ADL difficulties predominantly increased in older ones. As difficulties with ADL and IADL often correspond to how much help and hands-on-care an older person needs, our findings indicate an increased need for care services among Germans with diabetes. Our results contradict the assumption of a “dynamic equilibrium” that would have been applied if ADL and IADL difficulties had shifted from major to moderate disabilities. Instead, for type 2 diabetes, the findings are pointing toward morbidity expansion. Among persons without diabetes, by contrast, predicted probabilities of ADL and IADL difficulties remained almost unchanged for both genders. Thus, our results suggest a widening functional health divide between people without and with this disease to the disadvantage of the latter.

### Change in Comorbidity Rates and its Contribution to the Rise in ADL and IADL Difficulties among People with Diabetes

Recent studies indicate that prevalence of multimorbidity is rising globally (Singer et al., 2019) as well as in Germany (Tetzlaff et al., 2017). However, less is known whether comorbidities have also increased among people with diabetes in Germany and what consequences this might have on their functional health. In Germany, DMPs for type 2 diabetes were enrolled in the year 2003. Quality of care of diabetes patients may be expected to improve within DMPs as they implemented evidence-based clinical practice guidelines and educational and quality control measures (Renders et al., 2001; Stark et al., 2011). Data of the first 12 years of DMPs confirmed an improvement in the quality of care in Germany. For example, the prescription of metformin increased from 40.5% in 2004 to 54.1% in 2015, and the proportion of patients completing diabetic education increased within this period from 12.8% to 29.3%. However, no significant improvement was observed with regard to smoking status or BMI weight status. With respect to obesity (BMI ≥ 35), the percentage among individuals with diabetes even tended to increase over time (Mehring et al., 2017). Improvements as well as changes to the worse were also found by Du and colleagues (Du et al., 2015), who investigated alterations in type 2 diabetes care indicators in Germany based on two national health examination surveys conducted in the years 1997–1999 and 2008–2011. Significant improvements were found for several indicators such as HbA1c < 7, statin use, and diabetes-specific complications. However, similar to the findings by Mehring et al. (2017), they observed that current smoking rates did not change and obesity rose over time. The systematic literature review by Fuchs et al. (2014) also revealed no clear effect of DMPs on comorbidity, BMI, or quality of life. In addition, no beneficial effect of DMPs on mental health outcomes such as psychological well-being, anxiety, or depression could be determined.

In our study, we found that the number of persons with diabetes having at least two somatic comorbidities has been significantly growing in Germany between 2004 and 2015. Depression rates also tended to increase among individuals with diabetes while failing to reach statistical significance. A large body of studies has demonstrated that the coexistence of diabetes and depression has a synergistic effect on the risk of disability and other adverse health outcomes. For example, the study by Black and colleagues (Black et al., 2003) revealed that patients with diabetes and coexisting depression had a 4.1-fold increased risk of incident disability compared to a 1.7-fold increased risk among adults with diabetes only. In addition, we found a marked increase in obesity rates among women with diabetes over time, which is likewise associated with an elevated risk of disabilities (Chen & Guo, 2008).

Our decomposition analysis showed that the increase in obesity and depression rates as well as in somatic comorbidities accounted for the rise in ADL difficulties. With a different emphasis in men and women, all three indicators of comorbidity also contributed significantly to the rise in IADL difficulties. Hence, we found evidence that the increase in obesity, depression, and comorbid conditions accounted for the rise in disability among individuals with diabetes. Our findings suggest that while quality of care for these people enhanced over time, there is still room for improvements. Given the finding of the rising tendency of depression among persons with diabetes, more attention should be paid on treating comorbid depression. In addition, rising obesity rates among individuals with diabetes, found in previous research (Du et al., 2015; Mehring et al., 2017) and confirmed in our study, indicate that there is a need to improve the behavioral risk factor control in particular. Given that health practices are embedded in social structures (Crossley, 2004), the wider context within which lifestyle takes shape may not be left out of consideration.

### Limitations

One important limitation of our study is the definition of type 2 diabetes cases which was done only according to age of onset, using the age of onset over 30 years as the threshold for classifying type 2 diabetes cases. Although in adults above 30 years, type 1 diabetes accounts for less than 5% of all cases (Diaz-Valencia et al., 2015), misclassification cannot completely be ruled out. In addition, persons who are hospitalized and severely ill did not participate in the survey, which may be resulting in an underestimation of diabetes prevalence and related disability. Although diabetes is not a rare disease, the number of cases went down, in particular, after stratification by gender and age-groups. Thus, some subanalyses may lack sufficient statistical power to detect significant changes. In addition, we used the criterion “at least one ADL or IADL difficulty” as a threshold value for being classified as disabled. We have conducted a sensitivity analysis in which we set the criterion “at least two ADL or IADL difficulties” as threshold and found that the observed disability trend among persons with and without diabetes remained stable. However, by using a dichotomous variable, we did not account for the number of functional difficulties placed in the social context. In addition, it should be noted that the measure of IADL difficulties has slightly changed between waves 1 and 6, possibly affecting the results obtained. However, by focusing on “at least one ADL or IADL difficulty,” this potential source of bias was minimized. Furthermore, the list of chronic somatic conditions used as the basis for determining comorbidities among people with diabetes did not include all relevant diseases, and thus did not provide a complete picture of their physical diseases. Finally, we analyzed changes in diabetes between two points in time. In order to draw conclusions on disability trends, more time points need to be considered.

## Conclusions

Our findings lend support to the morbidity expansion hypothesis, indicating that not only diabetes prevalence but also proportions of ADL and IADL difficulties among individuals with diabetes increased over time. With different emphasis for men and women, the parallel rise in somatic comorbidities as well as in obesity and depression rates could be identified as drivers of this increase. Our findings suggest a growing demand of tertiary prevention among people with diabetes in Germany.
